# Supplementary figures and images for: A new insight into RecA filament regulation by RecX from the analysis of conformation-specific interactions
Source: eLife. 2022 Jun 22;11:e78409. doi: 10.7554/eLife.78409 (PMC9252578; doi:10.7554/eLife.78409)

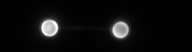

Supplement: Figure 4—source data 2. [file elife-78409-fig4-data2.zip › Single images revised/apo/4.tif]

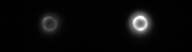

Supplement: Figure 4—source data 2. [file elife-78409-fig4-data2.zip › Single images revised/ATPgS/ATPgS_3.tif]

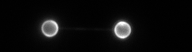

Supplement: Figure 4—source data 2. [file elife-78409-fig4-data2.zip › Single images revised/apo/1.tif]

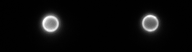

Supplement: Figure 4—source data 2. [file elife-78409-fig4-data2.zip › Single images revised/ATPgS/ATPgS_6.tif]

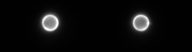

Supplement: Figure 4—source data 2. [file elife-78409-fig4-data2.zip › Single images revised/ATPgS/ATPgS_1.tif]

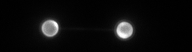

Supplement: Figure 4—source data 2. [file elife-78409-fig4-data2.zip › Single images revised/apo/2.tif]

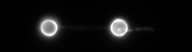

Supplement: Figure 4—source data 2. [file elife-78409-fig4-data2.zip › Single images revised/apo/6.tif]

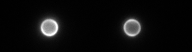

Supplement: Figure 4—source data 2. [file elife-78409-fig4-data2.zip › Single images revised/ATP/3.tif]

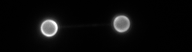

Supplement: Figure 4—source data 2. [file elife-78409-fig4-data2.zip › Single images revised/apo/3.tif]

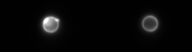

Supplement: Figure 4—source data 2. [file elife-78409-fig4-data2.zip › Single images revised/ATPgS/ATPgS_4.tif]

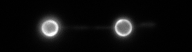

Supplement: Figure 4—source data 2. [file elife-78409-fig4-data2.zip › Single images revised/apo/5.tif]

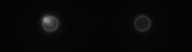

Supplement: Figure 4—source data 2. [file elife-78409-fig4-data2.zip › Single images revised/ATPgS/ATPgS_5.tif]

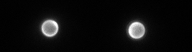

Supplement: Figure 4—source data 2. [file elife-78409-fig4-data2.zip › Single images revised/ATP/2.tif]

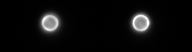

Supplement: Figure 4—source data 2. [file elife-78409-fig4-data2.zip › Single images revised/ATPgS/ATPgS_2.tif]

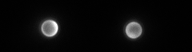

Supplement: Figure 4—source data 2. [file elife-78409-fig4-data2.zip › Single images revised/ATP/1.tif]
