# Supplementary material for: A new insight into RecA filament regulation by RecX from the analysis of conformation-specific interactions
Source: eLife. 2022 Jun 22;11:e78409. doi: 10.7554/eLife.78409 (PMC9252578; doi:10.7554/eLife.78409)
Supplement: Source code 1. [file elife-78409-code1.zip › 06-03-2022-RA-eLife-78409/ImageJ macros RecX_mNG.docx]

//open an image and apply rotation if needed to align beads parallel to horizontal axis

//background subtraction

makeRectangle(0, 0, 8, 52);

getRawStatistics(nPixels, mean, min, max, std, histogram);

makeRectangle(0, 0, 192, 52);

run("Subtract...", "value=mean");

//choosing central area for mean intensity analysis

//makeRectangle(83, 25, 11, 6); //apo

//makeRectangle(76, 25, 31, 6); //ATP

//makeRectangle(85, 20, 31, 6); //ATPgS

getRawStatistics(nPixels, mean, min, max, std, histogram);

print(mean);
